# Activation of Free Fatty Acid Receptor 4 Affects Intestinal Inflammation and Improves Colon Permeability in Mice

**DOI:** 10.3390/nu13082716

**Published:** 2021-08-06

**Authors:** Maciej Salaga, Adrian Bartoszek, Agata Binienda, Julia B. Krajewska, Adam Fabisiak, Paula Mosińska, Katarzyna Dziedziczak, Karolina Niewinna, Marcin Talar, Aleksandra Tarasiuk, Radzisław Kordek, Jakub Fichna

**Affiliations:** 1 Department of Biochemistry, Faculty of Medicine, Medical University of Lodz, Mazowiecka 6/8, 92-215 Lodz, Poland; salaga.maciej@gmail.com (M.S.); adrian.bartoszek@stud.umed.lodz.pl (A.B.); agata.binienda@gmail.com (A.B.); krajewska.julia@gmail.com (J.B.K.); adam.fabisiak@umed.lodz.pl (A.F.); paula.mosinska@gmail.com (P.M.); katarzyna.dziedziczak@umed.lodz.pl (K.D.); karolina.niewinna@umed.lodz.pl (K.N.); marcin.talar@umed.lodz.pl (M.T.); aleksandra.tarasiuk@umed.lodz.pl (A.T.); 2 Department of Digestive Tract Diseases, Medical University of Lodz, 92-215 Lodz, Poland; 3 Department of Pathology, Faculty of Medicine, Medical University of Lodz, 92-215 Lodz, Poland; radzislaw.kordek@umed.lodz.pl

**Keywords:** inflammatory bowel disease, free fatty acid receptors, intestinal permeability

## Abstract

Diet is considered an important trigger in inflammatory bowel diseases (IBD), as feeding habits can affect intestinal permeability and clearance of bacterial antigens, consequently influencing the immune system. Free fatty acid receptors (FFARs), expressed on the intestinal epithelial cells, belong to the family of luminal-facing receptors that are responsive to nutrients. The objective of this study was to characterize the anti-inflammatory activity and the effect on intestinal barrier function of synthetic FFAR agonists in mouse models of colitis. Therapeutic activity of GW9508 (FFAR1 agonist), 4-CMTB (FFAR2 agonist), AR420626 (FFAR3 agonist), and GSK137647 (FFAR4 agonist) was investigated in two models of semi-chronic colitis: induced by trinitrobenzenesulfonic acid (TNBS), mimicking Crohn’s disease, as well as induced by dextran sulfate sodium (DSS), which recapitulates ulcerative colitis in humans. Moreover, we assessed the influence of FFARs agonists on epithelial ion transport and measured the ion flow stimulated by forskolin and veratridine. Administration of FFAR4 agonist GSK137647 attenuated both TNBS-induced and DSS-induced colitis in mice, as indicated by macroscopic parameters and myeloperoxidase activity. The action of FFAR4 agonist GSK137647 was significantly blocked by pretreatment with selective FFAR4 antagonist AH7614. Moreover, FFAR1 and FFAR4 agonists reversed the increase in the colon permeability caused by inflammation. FFAR4 restored the tight junction genes expression in mouse colon. This is the first evaluation of the anti-inflammatory activity of selective FFAR agonists, showing that pharmacological intervention targeting FFAR4, which is a sensor of medium and long chain fatty acids, attenuates intestinal inflammation.

## 1. Introduction

Inflammatory bowel disease (IBD) is a group of conditions characterized by chronic, relapsing inflammation of the gastrointestinal (GI) tract, comprising ulcerative colitis (UC) and Crohn’s disease (CD) [1]. It is a heterogeneous complaint with contributions from genetic background, microbiota, and several environmental factors. Neither CD nor UC are fatal diseases, but both are very debilitating, with a broad range of symptoms, including abdominal pain, diarrhea, rectal bleeding, fever, vomiting, and weight loss. Consequently, the chronic nature of the symptoms leads to a reduction in the patient’s quality of life [2]. There is no cure for the disease, with only conservative treatment options available, such as anti-inflammatory drugs, steroids, immunosuppressants, or antibodies aiming at cytokines and integrins [3].

Carboxylic acids with an aliphatic tail, also referred to as free fatty acids (FFAs), are not only fundamental metabolic substrates for energy generation but also significant modulators of many cellular functions, including migration, proliferation, apoptosis, and production of reactive oxygen species, nitric oxide, eicosanoids, cytokines, and hormones [2]. FFAs are endogenous ligands of G protein-coupled receptors (GPCRs), referred to as free fatty acid receptors (FFARs). To date, four types of those receptors called FFAR1, FFAR2, FFAR3, and FFAR4, all belonging to the rhodopsin-like family of GPCRs, were identified [2]. FFAR2 and 3 are activated by short-chain FFAs (SCFAs), such as acetate and propionate, whereas FFAR1 and 4 respond to medium- and long-chain FFAs (LCFAs). FFAR1, FFAR2, and FFAR3 exhibit relatively high amino acid sequence similarity ranging from 34 to 52%, while FFAR4 manifests only a minor resemblance to FFAR1 [4]. Of note, FFAR4 is expressed in humans as long (BC101175, FFAR4L) and short (NM_181745, FFAR4S) isoforms that differ by 16 amino acids in the third intracellular loop of the former [5].

All types of FFARs are expressed in various parts of the GI tract. FFAR1 is present chiefly in pancreatic β cells, intestinal enteroendocrine cells: I-producing cholecystokinin (CCK) and L-releasing glucagon-like peptide 1 (GLP-1) and peptide YY (PYY) [2]. FFAR2 was shown to be expressed in gastric ghrelin cells and immune cells: neutrophils, eosinophils, monocytes, and intestinal T reg cells [2]. FFAR3 was found in K cells on enteric neurons and sympathetic ganglia. FFAR4, being present in intestinal enteroendocrine cells (I, K, L), has a similar expression to FFAR1 [2]. Furthermore, expression of FFAR4L was detected exclusively in the colon, while the FFAR4S was present in various organs [6].

At the molecular level, activation of FFARs leads to release of Ca^2+^ and extracellular signal-regulated kinase 1/2 (ERK1/2) phosphorylation. In addition, FFAR1 and 2 were showed to recruit β-arrestin 2 [7,8]. FFAR4L activates only β-arrestin pathway whereas FFAR4S acts through both Gq/11 and β-arrestin [9]. Therefore, stimulation of FFARs leads to various changes in the body, including modulation of inflammation, and release of hormones like GLP-1, GIP, and insulin [10,11]. Reports revealed the likely use of FFAs in the treatment of type 2 diabetes, asthma, cardiovascular diseases, and metabolic syndrome. However, the impact of FFARs on intestinal inflammation has not been fully explored.

Studies on FFAR1 and 4 activation showed their anti-inflammatory potential in cell lines of GI origin [12,13]. Lee et al. [14] showed that esters of hydroxyl stearic acid attenuate colitis in mice through a mechanism that may involve FFAR4. Results on FFAR2 in the context of GI inflammation are contradictory. Agus et al. [15] demonstrated that mice treated with a FFAR2 agonist were protected against dextran sulfate sodium (DSS)-induced colitis. Contrary outcomes were presented by two other research groups whose investigations showed that FFAR2 KO mice were preserved against DSS and 2,4,6-trinitrobenzenesulfonic acid solution (TNBS)-induced colitis [16,17]. Similar results were obtained for FFAR3 [17].

Thus, in search for new targets and particles with potential therapeutic value, we tested the hypothesis that activation of FFARs by synthetic agonists alleviates colitis and improves colonic barrier function.

## 2. Materials and Methods

### 2.1. Cell Line Culture

The RAW264.7 murine macrophages (ATCC: TIB-71) were cultured in Dulbecco’s Modified Eagle Medium (Gibco) supplemented with 10% bovine calf serum (BCS), 2 mM Alanine-Glutamine, 0.5% penicillin-streptomycin (P/S), 1 mM sodium pyruvate, and 25 mM HEPES. Cells were grown in an atmosphere of 5% CO_2_ at 37 °C. The cells were passaged after reaching approx. 80% confluence.

The Caco-2 human colonic epithelial cell line was obtained from the American Type Culture Collection (ATCC: HTB-37). The cells were cultured in Eagle’s Minimal Essential Medium (Gibco) with addition of 20% fetal bovine serum (FBS), 4 mM Ala-Gln, 0.1 mM nonessential amino acids, 0.5% P/S, 1 mM sodium pyruvate, and 25 mM HEPES. Cells were grown in an atmosphere of 5% CO_2_ at 37 °C. The cells were trypsinized and passaged after reaching approx. 80% confluence.

### 2.2. Cytotoxicity Assessment

The toxicity in RAW264.7 cells was evaluated with neutral-red uptake (NRU) test, which characterizes the lysosomal activity of viable cells [18]. The cells were placed in 96-well plates (20,000 cells/well). FFAR agonists in the concentrations of 10, 20, 50, and 80 µM were chosen based on the preliminary experiments (data not shown), and budesonide was applied at the concentration of 10 µM. Cells were exposed to the compounds for 48 h before the measurement of cytotoxicity. Then, cells were incubated for 1 h with 100 μL/well of 0.05 mg/mL NR solution in culture medium. Next, cells were washed with phosphate-buffer saline (PBS, pH 7.4) and 100 μL/well of 40% ethanol, and 10% acetic acid in water was used to dissolve the dye. After 10 min of shaking, the absorbance was measured at 540 nm in a microplate reader (iMARK Microplate Reader, Biorad, Hertfordshire, UK). Data were expressed as a percentage of cells without any treatment.

The cytotoxicity in Caco-2 cell line was evaluated with the MTT assay [19]. Briefly, cells were seeded on a 96-well plate at the density of 20,000 cells/well and incubated for 24 h with FFAR agonists in the following concentrations: 10, 30, 50, 100, and 200 µM. Budesonide was applied at the concentration of 10 µM. Then, 20 µL of MTT solution (5 mg/mL) was added to each well and incubated for 2 h. Next, medium was replaced with 100 µL of dimethyl sulfoxide (DMSO), and the plate was shaken for 15 min. Optical density was measured by microplate reader (iMARK Microplate Reader, Biorad, Hertfordshire, UK) at 595 nm. Data were expressed as a percentage of cells without any treatment.

### 2.3. Griess Assay (Measurement of Nitrite Secreted by RAW264.7 Cells)

The cells were placed in 96-well plates (20,000 cells/well) and incubated for 24 h with standard culture medium (control) or medium with 1 μg/mL lipopolysaccharide (LPS) with or without FFAR agonists (in the same concentrations as for cytotoxicity assessment) or budesonide. After 24 h of incubation, LPS was removed from the culture by replacing the media with new solutions containing FFAR agonists or budesonide but without LPS. After another 24 h, 100 µL of cell culture supernatant was mixed with 100 µL of Griess reagent water solution (40 mg/mL), and the mixture was kept in the dark for 15 min. Next, the absorbance was read at 540 nm. Nitrite concentration was expressed as a percentage of the cells treated with LPS only.

### 2.4. In Vitro Model of Cytokine and LPS-Induced Inflammation

The model was adapted from the study by Van De Walle et al. [20]. Briefly, the Caco-2 cells were seeded on a 24-well plate at the density of 100,000 cells/well and incubated for 24 h with medium. Then, a mixture of tumor necrosis factor α (TNFα; 50 ng/mL), interleukin 1β (IL-1β; 25 ng/mL), interferon γ (IFNγ; 50 ng/mL), and LPS (100 ng/mL) was added to each well. Experimental groups were additionally treated with FFAR agonists in concentrations selected based on the outcomes of cytotoxicity assessment: FFAR1 100 µM, FFAR2 30 µM, FFAR3 50 µM, and FFAR4 30 µM. After 12 h of incubation, cell culture medium was harvested and used for IL-6 measurement with a human IL-6 ELISA kit (cat. No. 950.030.096, Diaclone, France) according to manufacturer instructions. Outcomes were expressed as a percentage of cells treated with a mixture of cytokines and LPS only.

### 2.5. Animals

We used male C57BL/6 mice (Mossakowski Medical Research Center Polish Academy of Sciences in Warsaw, Poland) weighing 22–26 g (6–8 weeks of age). Mice were kept at 22 °C and under a 12-h light/dark cycle in sawdust-lined plastic cages. Chow pellets and tap water were provided ad libitum. All animal protocols were approved by the Medical University of Lodz Animal Care Committee (Protocol 34/ŁB 96/2018) and complied with the European Communities Council Directive of 22 September 2010 the EU (2010/63/EU).

### 2.6. Induction of Colitis

#### 2.6.1. TNBS Model

Colitis was evoked by intracolonic (i.c.) instillation of TNBS [21]. After weighing, animals were anesthetized with 1% isoflurane (Baxter Healthcare Corp., Naperville, IL, USA). Solution of 4% TNBS and 30% ethanol in saline (0.1 mL) was injected through a catheter 3 cm proximally from the rectum. Then, to ensure thorough distribution of the solution in the colon, animals were held in an inclined position for approx. 1 min. Vehicle alone was administered in the control group (30% ethanol in saline; TNBS replaced with equivolume water). Our previous experiments demonstrated that 4% TNBS is sufficient to induce colitis, resulting in development of clinical symptoms at the macro- and microscopic level as well as biochemical changes characteristic for intestinal inflammation.

#### 2.6.2. DSS Model

Mice were randomized, and colonic inflammation was induced, as described before [22]. On day 0 of the experiment, DSS (4% *w*/*v*; molecular weight 40,000 (MP Biomedicals, Aurora, OH, USA, Lot No. 5237K)) was added to drinking water. On day 5, DSS solution was replaced with tap water. Animals were sacrificed on day 7. Control animals were receiving tap water throughout the whole experiment.

Our previous experiments demonstrated that 4% DSS is sufficient to induce colitis, resulting in development of clinical symptoms at the macro- and microscopic level as well as biochemical changes characteristic for intestinal inflammation.

### 2.7. Evaluation of Colonic Damage

#### 2.7.1. TNBS-Induced Colitis

Disease parameters were evaluated on day 7 following administration of TNBS. Mice were sacrificed, and the colon was rapidly removed and, upon opening, rinsed with PBS. Macroscopic colonic damage was determined by an semiquantitative scoring based on adding individual scores for ulcer, shortening of the colon, colonic wall thickness, and presence of hemorrhage, fecal blood, and diarrhea. The following system was used to score ulceration and shortening of the colon: ulcer: 0.5 points for each 0.5 cm; shortening of the colon: 1 point for >15%, 2 points for >25% (relative to an average length of the colon in control animals equal to 8.59 ± 0.33 cm; *n* = 5). Colonic wall thickness of n mm equaled n scoring points. Hemorrhage, fecal blood, or diarrhea increased the score by 1 point.

#### 2.7.2. DSS-Induced Colitis

On day 7 of the experiment, mice were euthanized, and the entire colon was excised and weighed with fecal content. Macroscopic damage score was calculated based on the stool consistency (0—normal, well-shaped fecal pellets; 3—diarrhea), colon epithelial damage (0–3), and colon length and weight scores, calculated as a loss of each parameter relative to the control group (0 points, ≤5% weight/length loss; 1 point, 5–14% weight/length loss; 2 points, 15–24% weight/length loss; 3 points, 25–35% weight/length loss; and 4 points, ≥35% weight/length loss). Total score = 0 means no inflammation. Fecal blood added 1 score to the total macroscopic damage.

### 2.8. Pharmacological Treatments

To assess therapeutic activity of FFARs, in both DSS and TNBS model, mice were injected intraperitoneally (i.p) twice daily for 4 consecutive days (on days 3–6). The following agents were used at the dose 1 mg/kg bw: GW9508 (FFAR1 agonist), 4-CMTB (FFAR2 agonist), AR420626 (FFAR3 agonist), and GSK137647 (FFAR4 agonist). AH7614 (FFAR4 antagonist) was administered at the dose of 5 mg/kg bw.

### 2.9. Determination of Tissue Myeloperoxidase Activity

MPO activity was measured according to the protocol described earlier [21]. Briefly, 1-cm segments of the colon were weighed, homogenized in hexadecyltrimethylammonium bromide (HTAB) buffer, and centrifuged (13,200× *g*, 15 min). Then, 7 μL of supernatants were added to each well on a 96-well plate containing 200 μL of 500 mM potassium phosphate buffer supplemented with 0.167 mg/mL of O-dianisidine hydrochloride and 0.05 μL of 1% H_2_O_2_. Absorbance (450 nm; triplicate) was measured 0, 30, and 60 s after the initiation of reaction. MPO activity was expressed in milliunits per gram of wet tissue, 1 unit being the quantity of enzyme able to convert 1 μmol of H_2_O_2_ to water in 1 min at room temperature (RT). Standard curve of MPO activity was made with purified peroxidase enzyme.

### 2.10. Immunohistochemistry

For immunohistochemical (IHC) staining, the method described by Sadej et al. [23] was used. Briefly, slides were washed in PBS followed, by quenching of endogenous peroxidase activity with PBS containing 0.6% (*v*/*v*) H_2_O_2_ and 10% (*v*/*v*) methanol for 30 min at RT. Next, slides were washed and immersed in blocking solution/antibody diluent containing 2% (*w*/*v*) bovine serum albumin (Sigma-Aldrich, St. Louis, MO, USA), 5% (*v*/*v*) goat serum (Dako, Carpinteria, CA, USA), and 0.2% (*v*/*v*) Triton X-100 in PBS for 1 h. The sections were incubated overnight at 4°C with primary antibodies to visualize FFAR1 (AFR-001, 1:200; Alomone Labs, Jerusalem, Israel), FFAR2 (AFR-032; 1:500; Alomone Labs, Jerusalem, Israel), FFAR3 (PA5-97747; dil. 1:200; Thermo Scientific, Rockford, IL, USA), or FFAR4 (NBP1-00858; 1:2000; Bio-Techne, Warsaw, Poland).

The following day, slides were washed extensively for 3 × 10 min in PBS and further incubated with the appropriate secondary antibody for 1.5 h at RT. After another round of washing, slides were incubated with an avidin–biotin complex reagent (1:200; Vector Laboratories, Burlingame, CA, USA) for 1 h at RT. The staining was visualized by incubating the slides with 0.1% (*w*/*v*) 3,3-diaminobenzidine and 0.03% (*v*/*v*) H_2_O_2_ in Tris-HCl, pH 7.6. The reaction was terminated after approx. 4–5 min. Then slides were dehydrated through a graded series of ethanol, cleared in xylene, and cover-slipped in Pertex mounting medium (HistoLab, Göteborg, Sweden). Evaluation of the staining was performed in the computerized Axo Imager Carl Zeiss light microscope. To quantify the images, we utilized Fiji software. Spots of FFAR 1–4 staining were separated on the basis of the hue, the saturation, and the brightness. Pixel coverage of the FFARs staining was calculated for epithelium areas. We applied the same cut-off levels in all the analyzed pictures selected to quantify FFARs in our experiment. The amount of FFARs was presented as a surface-area coverage (%).

### 2.11. Ex Vivo Measurement of Epithelial Ion Transport

Epithelial ion transport was evaluated according to the method described previously [24]. Briefly, sections (around 1 cm) of the distal large intestine were obtained and immediately positioned in Ussing chamber (Physiologic Instruments, Inc., San Diego, CA, USA) with 6 mL of Krebs solution of the following constitution (mM): NaCl, 115; KH_2_ PO_4_, 2; MgCl_2_, 2.4; NaHCO_3_, 25; KCl, 8; and CaCl_2_, 1.3. The solution was saturated with 95% O_2_ and 5% CO_2_ and supplemented with either glucose (10 mM) or mannitol (10 mM) by the basolateral and mucosal side, respectively. The bath temperature was kept at 37 °C. The exposed area of the tissue was 0.3 cm^2^. Tissues were voltage clamped to zero, using the WPI EVC-4000 voltage clamp apparatus (World Precision Instruments, Sarasota, FL, USA) with Ag/AgCl electrode and 3 M KCl agar bridge. Once a stable baseline in short circuit current (Isc, mA/cm^2^) was reached (15–30 min), we tested FFAR agonist: GW9508 (10^−5^ M, FFAR1 agonist), 4-CMTB (10^−5^ M, FFAR2 agonist), AR 420626 (10^−5^ M, FFAR3 agonist), and GSK 137647 (10^−5^ M, FFAR4 agonist), dissolved in DMSO or an equivalent volume of vehicle (DMSO, final concentration: 0.1%) pipetted to the basolateral side of the chamber. After 10 min, specimens were challenged with either forskolin (FSK) (10^−5^ M, cAMP-dependent secretagogue) or veratridine (VER) (3 × 10^−5^ M, voltage-dependent Na^+^ channel activator). For each challenge, the peak change in Isc (∆Isc) was established.

### 2.12. Ex Vivo Assessment of Intestinal Permeability

For measurements of intestinal permeability, sections of the mouse large intestine were mounted in Ussing chambers (0.3 cm^2^ opening), and mucosal and serosal sides were exposed to 6 mL of oxygenated Krebs buffer. Mannitol and glucose (both 10 mmol) were introduced, respectively, and the tissues were allowed to incubate for 15 min. The experiment was started by replacing the buffer in the mucosal compartment with 10 mL Krebs buffer with fluorescein isothiocyanate dextran (FITC-dextran; Sigma-Aldrich) at a concentration of 1 mg/mL. Then, compartments were protected from light by aluminum foil. Fluorescence in the serosal chamber was measured instantly to establish baseline fluorescence. Specimens were taken from the serosal chambers at 20-min intervals for a total period of 2 h, and FITC-dextran concentration was measured at an excitation wavelength of 494 nm and emission wave-length of 521 nm.

### 2.13. RNA Isolation, Reverse Transcription and qPCR

In brief, RNA was sequestered from the distal sections of large intestine (weighing 20–30 mg) from both healthy and DSS-treated animals, in accordance with the manufacturer’s protocol using Total RNA Mini Plus kit (A&A Biotechnology, Gdansk, Poland). RNA was eluted from ion exchange columns by diethyl pyrocarbonate (DEPC)-treated water (40 μL). Then, total RNA was purified using Lithium Chloride Precipitation Solution in accordance with the manufacturer’s protocol (Life Technologies, Carlsbad, CA, USA). The purity and amount of isolated RNA was estimated using Colibri Microvolume Spectrophotometer (Biocompare, San Francisco, CA, USA). Total RNA (2 μg) was transcribed to cDNA with high-capacity Reverse Transcriptase Kit (Life Technologies, Carlsbad, CA, USA) in accordance with the manufacturer’s protocol. Quantitative assay of the expression was executed using fluorescently labeled probes (Life Technologies, Carlsbad, CA, USA): OCLN (Mm00500912_m1), CLDN1 (Mm00516701_m1), CLDN2 (Mm00516703_s1), CLDN3 (Mm00515499_s1), CLDN4 (Mm00515514_s1), CLDN7 (Mm00516817_m1), CLDN10 (Mm01226326_g1), CLDN12 (Mm01316510_m1), and GAPDH (Mm99999915_g1) as endogenous control on Mastercycler S realplex 4 (Eppendorf, Hamburg, Germany) using TaqMan Gene Expression Master Mix (Life Technologies, Carlsbad, CA, USA) according to the manufacturer’s protocol. All experiments were conducted in triplicate. The threshold cycle (Ct) values for studied genes were normalized to Ct values received for GADPH. The relative quantity of mRNA copies was calculated using the equation: 2^−ΔCt^ × 1000.

### 2.14. Western Blot

Tissue samples and cell culture samples were incubated with the mammalian cell lysis buffer (50 mM Tris−HCl, pH 7.5; 1 mM EDTA, 150 mM NaCl; 0.1% SDS; 0.5% deoxycholic acid; and 1% Igepal CA-630; Sigma-Aldrich, Poznan, Poland) containing protease-inhibitor cocktail. Afterwards, samples were homogenized using Precellys Evolution Homogenizer (Bertin Instruments, Paris, France) and centrifuged at 15,000 g for 15 min at 4 °C to remove the debris. Total protein concentration was measured using the Pierce 660 nm protein assay (Thermo Scientific, Rockford, IL, USA). Electrophoresis was performed using precast 4%–20% SDS-PAGE gel (Bio-Rad, Warsaw, Poland) in the buffer containing 0.1% SDS, 192 mM glycine, 25 mM Tris, pH 8.3. Separated proteins were transferred onto PVDF membranes (pore size, 0.45 μm; Life Technologies, Carlsbad, CA, USA) using a semi-dry system with transfer buffer containing 20% (*v*/*v*) methanol, 192 mM glycine, and 25 mM Tris, pH 8.3. The PVDF membranes were then blocked at RT for 1 h in 5% non-fat dry milk in phosphate buffered saline (PBS) with Tween 20 (PBST; PBS, 0.1% Tween 20). Then, membranes were incubated for 80 min at RT with specific primary antibodies diluted in 1% non-fat dry milk in PBST for immunodetection of the proteins of interest. The primary rabbit anti-mouse FFAR1 polyclonal antibody (AFR-001; dil. 1:1000; Alomone Labs, Jerusalem, Israel), FFAR2 polyclonal antibody (AFR-032, dil. 1:1000; Alomone Labs, Jerusalem, Israel), FFAR3 polyclonal antibody (PA5-97747; dil. 1:1000; Thermo Scientific, Rockford, IL, USA), FFAR4 polyclonal antibody (E-AB-31576; dil. 1:1000; Elabscience, Wuhan, China), and mouse anti-glycerylaldehyde-3-phosphate dehydrogenase (β-actin; sc-47778; dil. 1:1000; Santa Cruz Biotechnology, Dallas, TX, USA) were used. After the washing using PBST (5 times, 3 min), membranes were incubated with appropriate secondary antibodies for 1 hat RT. Then bands were visualized using Super Signal west pico western blotting substrate (Thermo Scientific, Rockford, IL, USA) as a substrate for the localization of HRP activity. Qualitative and quantitative analysis was performed by measuring integrated optical density (IOD) by ImageLab v5.2.1 for WindowsTM program (Bio-Rad SA, Warsaw, Poland).

### 2.15. Reagents and Drugs

All drugs and reagents, unless otherwise stated, were purchased from Sigma-Aldrich (Poznan, Poland). FFAR ligands were purchased from Bio-Techne (Warsaw, Poland).

### 2.16. Statistics

Statistical analyses were performed using Prism v9.0 (GraphPad Software Inc., La Jolla, CA, USA). The data are expressed as means ± SEM. Normality of data was evaluated with Shapiro–Wilk test. Student t-test or one-way ANOVA followed by Newman–Keuls post-hoc test was used for analysis. *p* values < 0.05 were considered statistically significant.

## 3. Results

### 3.1. FFAR1, FFAR3, and FFAR4 Agonists Reduced the Production of Nitric Oxide (NO) in Macrophages

**Figure 1:**
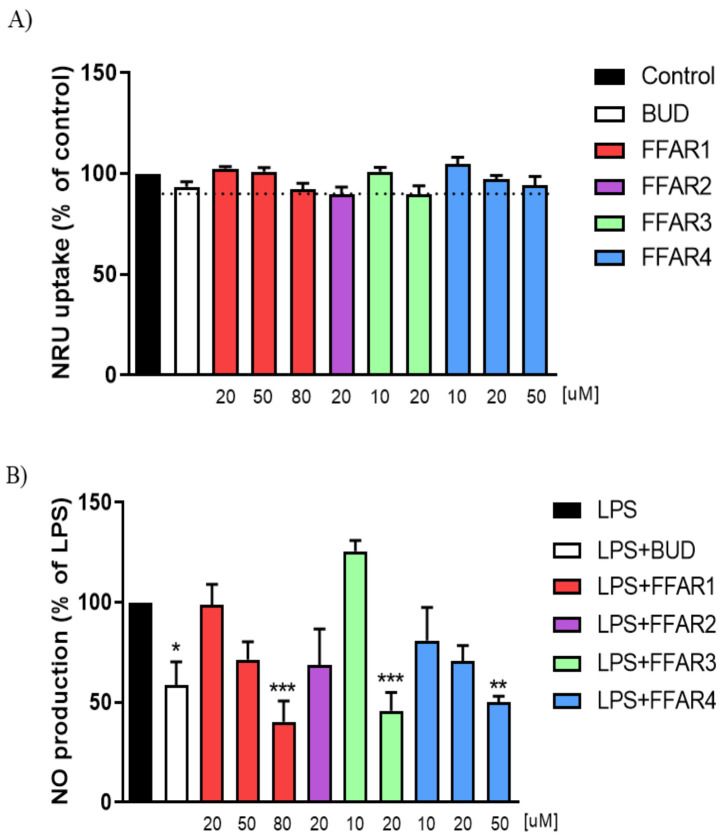

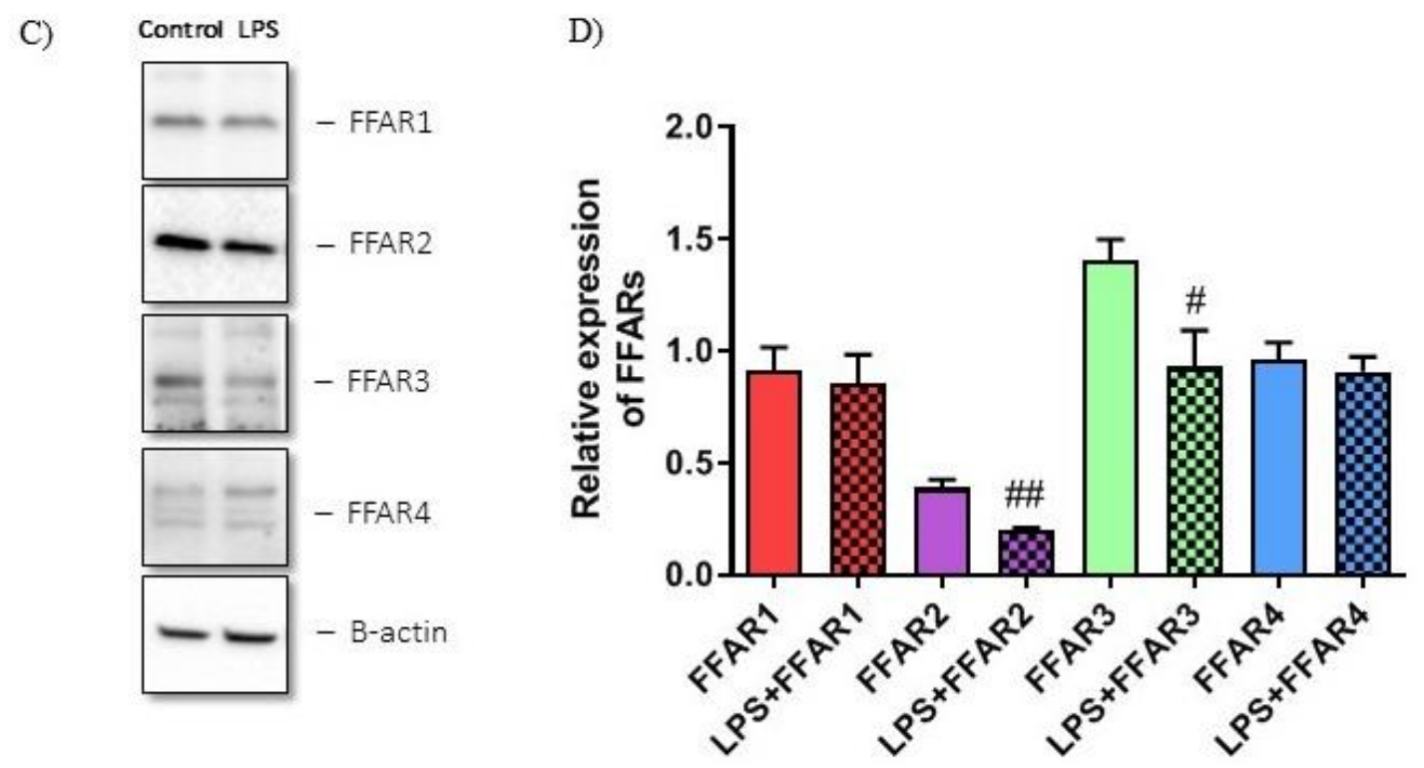
The effect of FFAR agonists on RAW264.7 macrophages in vitro. Figure shows data for cell survival measured by neutral-red (NRU) uptake after treatment with various concentrations of respective agonists (**A**), nitric oxide (NO) secretion after 24 h stimulation with lipopolysaccharide (LPS), and treatment with various concentrations of respective agonists (**B**). Representative western blots demonstrating the expression of all analyzed proteins in the whole cell lysates (**C**) and changes in the protein concentration of FFARs after 24 h exposure to LPS (**D**). Data represent mean ± SEM of *n* = 2–4 independent experiments (NRU, NO), and *n* = 5 samples per group (WB). Data were analyzed with one-way ANOVA, followed by Newman–Keuls post-hoc test (NRU, NO) and Student’s *t*-test (WB) * *p* < 0.05, ** *p* < 0.01, *** *p* < 0.001, as compared to LPS-treated group; ^#^ *p* < 0.05, ^##^ *p* < 0.01, as compared to non-LPS-treated group. BUD, budesonide.

In order to assess the anti-inflammatory effect of FFAR agonists, we used RAW264.7 macrophages treated with LPS. We observed that FFAR1 agonist GW9508 (80 µM), FFAR3 agonist AR420626 (20 µM), and FFAR4 agonist GSK137647 (50 µM) significantly reduced the level of NO secreted to the culture medium without affecting cell viability (Figure 1A,B). The effect of FFAR agonists was more potent than the reference drug budesonide (BUD) at a concentration of 10 µM. Investigation into expression of FFAR1, FFAR2, FFAR3, and FFAR4 in macrophages revealed that treatment with LPS significantly downregulated FFAR2 and FFAR3 as compared to control (Figure 1C,D).

### 3.2. Stimulation of FFAR4 Alleviated Response to Inflammatory Stimuli In Vitro

**Figure 2:**
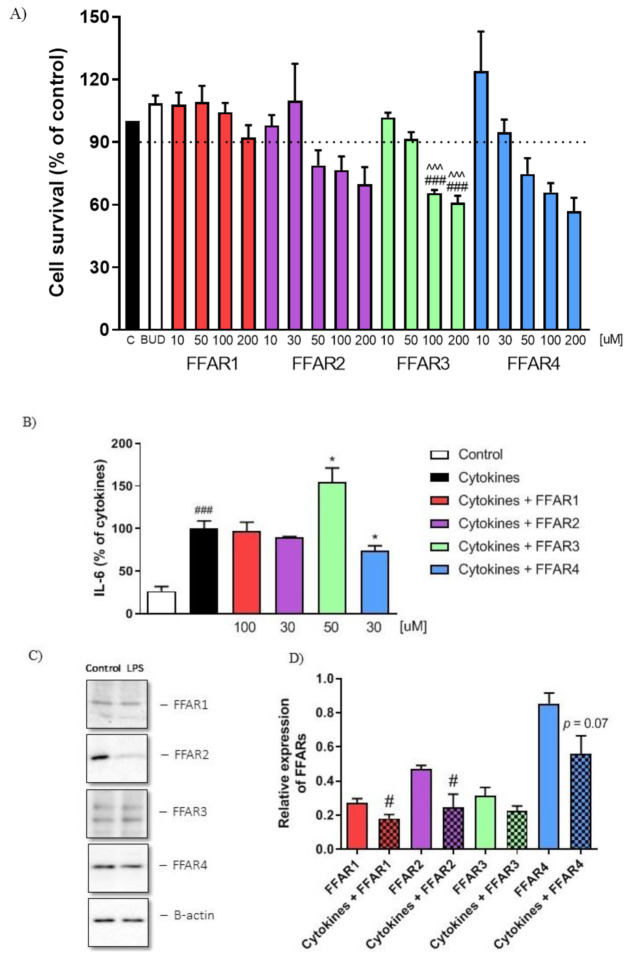
The effect of FFAR agonists on Caco-2 cells in vitro. Figure shows data for cell survival measured by MTT test after treatment with increasing concentration of FFAR agonists (**A**), secretion of IL-6 in response to pro-inflammatory cytokines and LPS, and treatment with respective agonists (**B**). Representative western blots demonstrating the expression of all analyzed proteins in the whole cell lysates (**C**) and changes in the expression of FFARs after exposure to pro-inflammatory cytokines and LPS (**D**). Data represent mean ± SEM of *n* = 2–4 independent experiments (MTT, ELISA), and *n* = 3 samples per group (WB). Data were analyzed with one-way ANOVA, followed by Newman–Keuls post-hoc test (cytotoxicity, IL6) and Student’s *t*-test (WB) * *p* < 0.05, as compared to cytokine-treated group; ^#^ *p* < 0.05, ^###^ *p* < 0.001 as compared to control or non-cytokine-treated group, ^^^^^ *p* < 0.001 as compared to budesonide-treated group.

To further characterize the effect of FFAR agonists on the inflammatory response, we measured the secretion of IL-6 in cytokine (mixture of TNFα, IL-1β, IFNγ) and LPS-treated Caco-2 cells. First, we assessed the cytotoxicity of all compounds and selected concentrations that do not affect cell viability (Figure 2A). We found that only FFAR4 agonist GSK137647 (30 µM) significantly reduced IL-6 secretion in response to the mixture of cytokines. On the other hand, FFAR3 agonist AR420626 (50 µM) significantly enhanced secretion of IL-6 (Figure 2B). Investigation into expression of FFARs in Caco-2 cells revealed that treatment with cytokines downregulated FFAR1, FFAR2, and FFAR4 as compared to control (Figure 2C,D).

### 3.3. FFAR 4 Agonist Alleviated Colitis in TNBS- and DSS-Treated Mice

**Figure 3:**
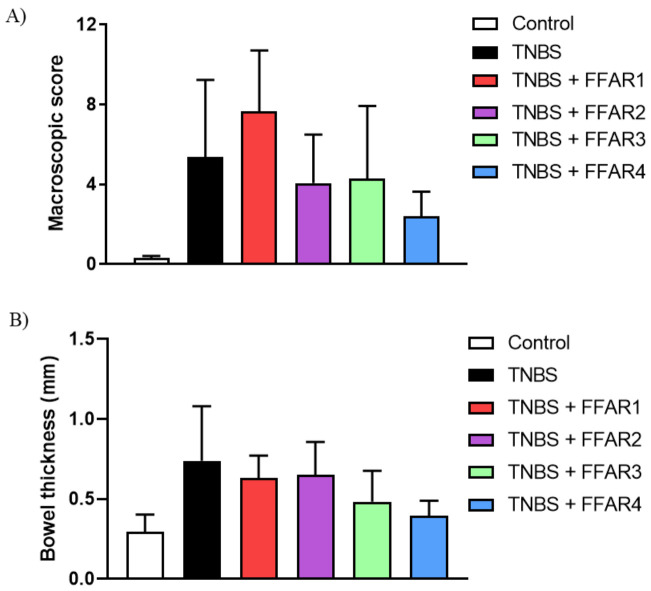

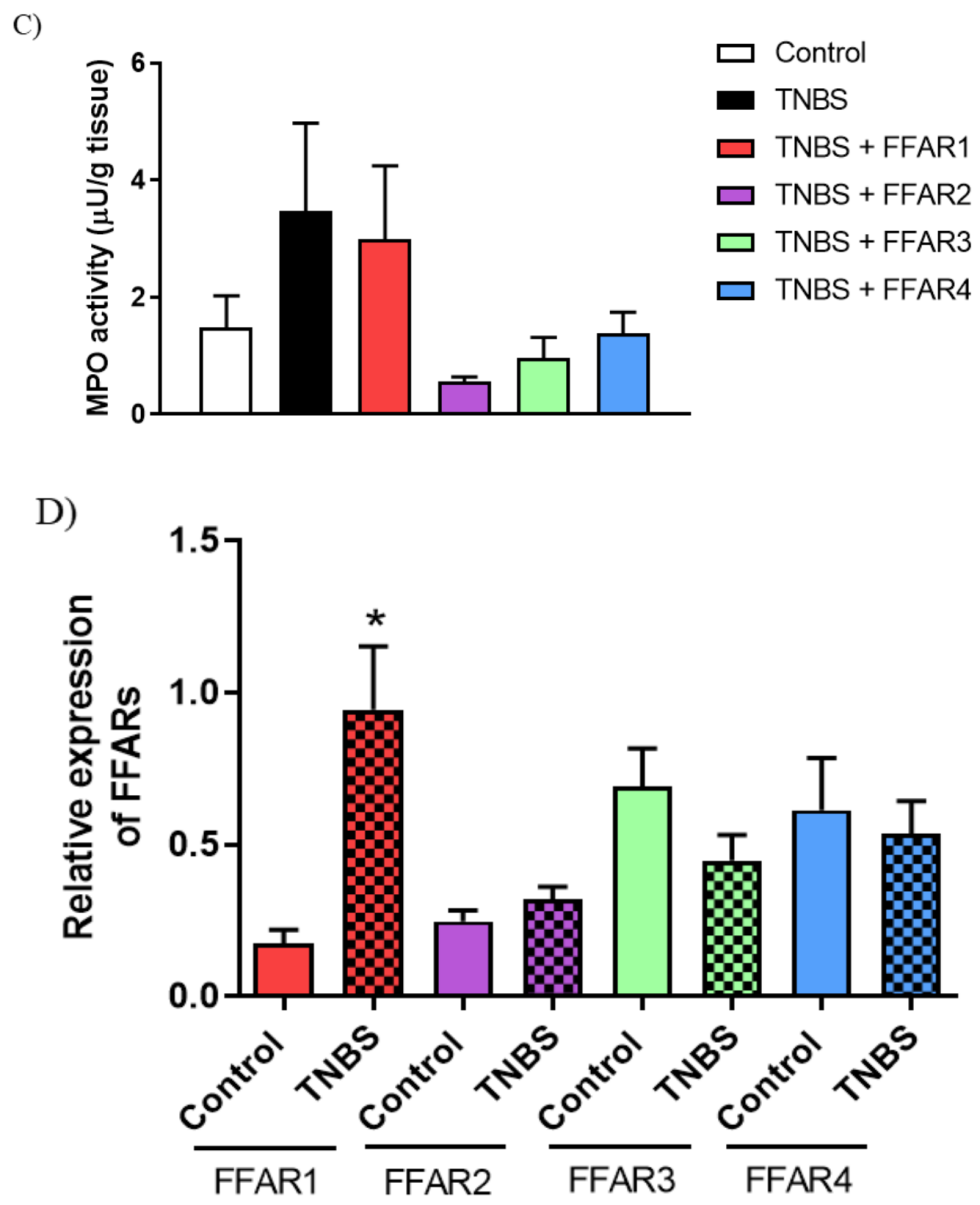
The effect of FFAR agonists administered at the dose of 1 mg/kg bw i.p. twice daily on TNBS-induced colitis in mice. Figure shows data for macroscopic damage score (**A**), bowel thickness (**B**), MPO activity (**C**), and relative expression at the protein level of FFARs in the colon of mice exposed to TNBS (**D**). Data represent mean ± SEM of *n* = 4–5 animals per group. Data were analyzed with one-way ANOVA, followed by Newman–Keuls post-hoc test (macroscopic score, bowel thickness, MPO) and Student’s *t*-test (expression) * *p* < 0.05, as compared to control group.

To evaluate the anti-inflammatory effect of FFAR agonists in the mouse GI tract, first, we used a well-established mouse model of GI inflammation induced by TNBS. The i.c. injection of TNBS resulted in reproducible colitis in mice, manifested by increased macroscopic colon-damage score and elevated MPO activity. We did not observe a significant decrease in macroscopic score, bowel thickness, and MPO activity after treatment with FFAR agonists. However, there was a clear, although not significant, anti-inflammatory effect of FFAR4 agonist GSK137647 (1 mg/kg bw, i.p., twice daily) (Figure 3A–C). Analysis of FFAR expression in the mouse colon revealed a significant increase of FFAR1 but not FFAR2–4 during inflammation (Figure 3D).

**Figure 4:**
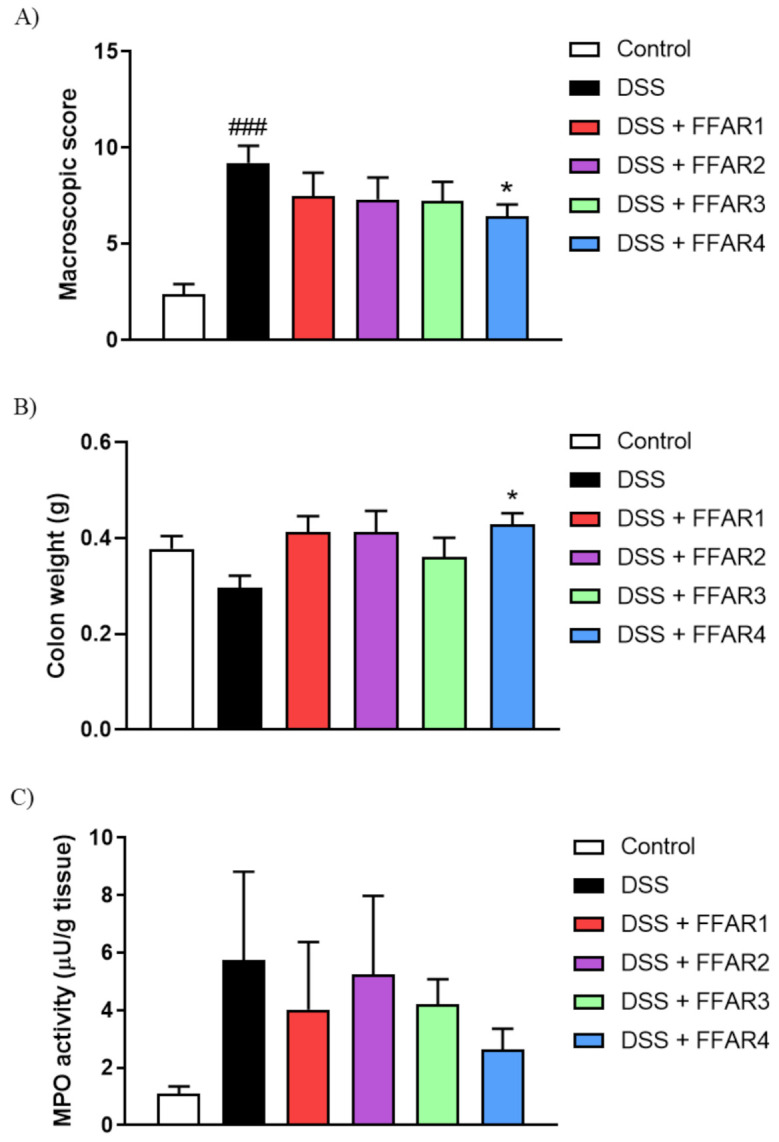

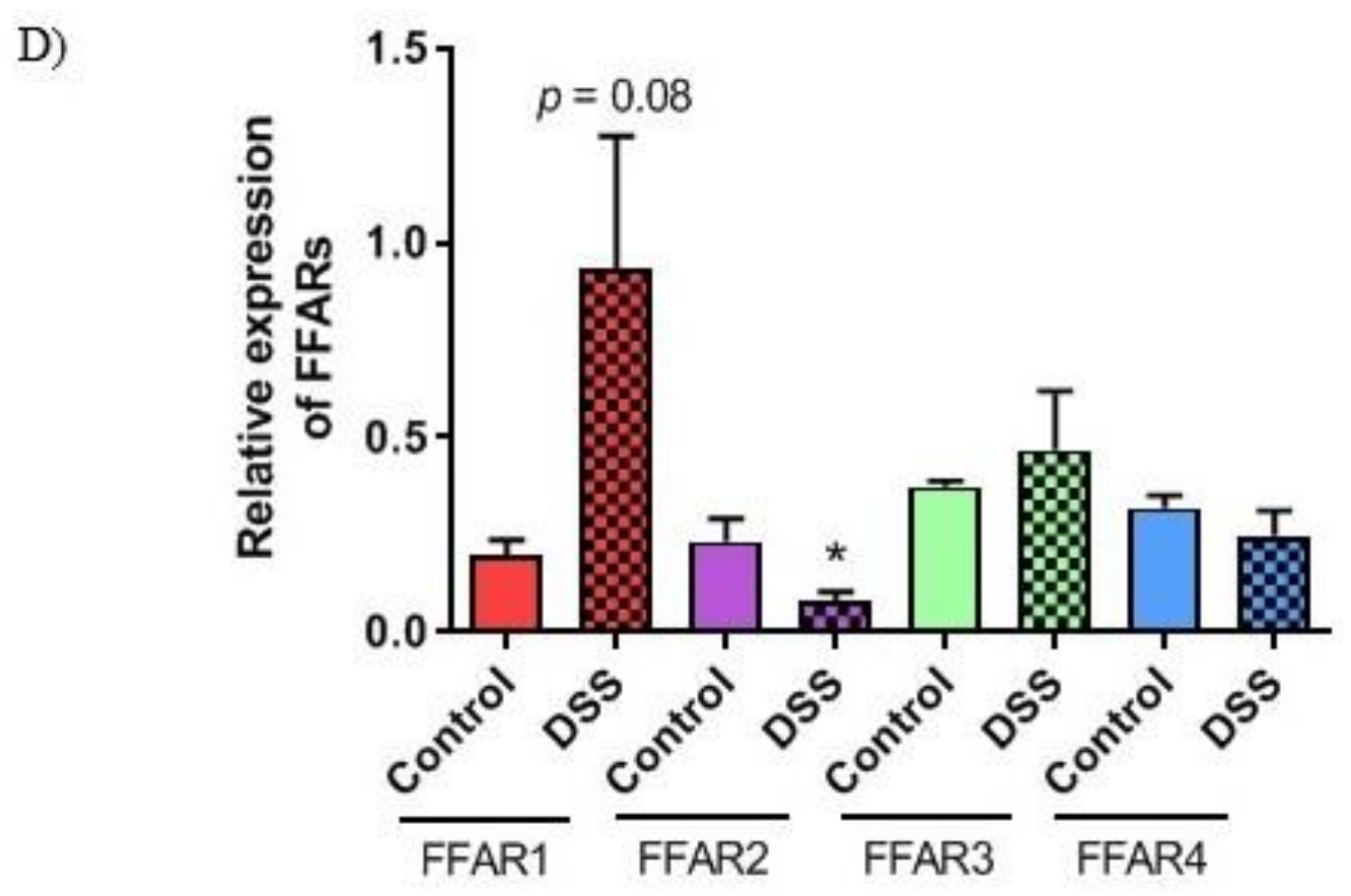
The effect of FFAR agonists administered at the dose of 1 mg/kg bw i.p. twice daily on DSS-induced colitis in mice. Figure shows data for macroscopic damage score (**A**), colon weight (**B**), MPO activity (**C**), and relative expression at the protein level of FFARs in the colon of mice exposed to DSS (**D**). Data represent mean ± SEM of *n* = 5–9 animals per group. Data were analyzed with one-way ANOVA, followed by Newman–Keuls post-hoc test (macroscopic score, colon weight, MPO) and Student’s *t*-test (expression) ^###^ *p* < 0.001, as compared to control group; * *p* < 0.05, as compared to DSS-treated group.

Additionally, we characterized the anti-inflammatory potential of FFAR agonists in the mouse model of UC induced by DSS. Animals treated with 4% DSS in drinking water developed severe colitis, characterized by increased macroscopic colon damage score, reduced colon weight, and elevated MPO activity (Figure 4).

The i.p. administration of FFAR4 agonist GSK137647 (1 mg/kg bw, twice daily) reversed colonic injury induced by DSS, as shown in Figure 4A–C.

Moreover, we observed that during DSS-induced colitis, expression of FFAR1 was significantly increased, while FFAR2 decreased in the colon (Figure 4D).

**Figure A1:**
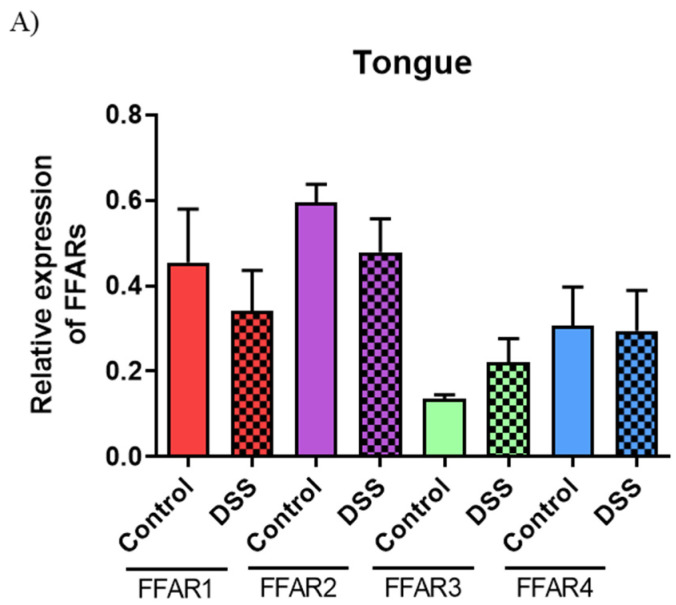

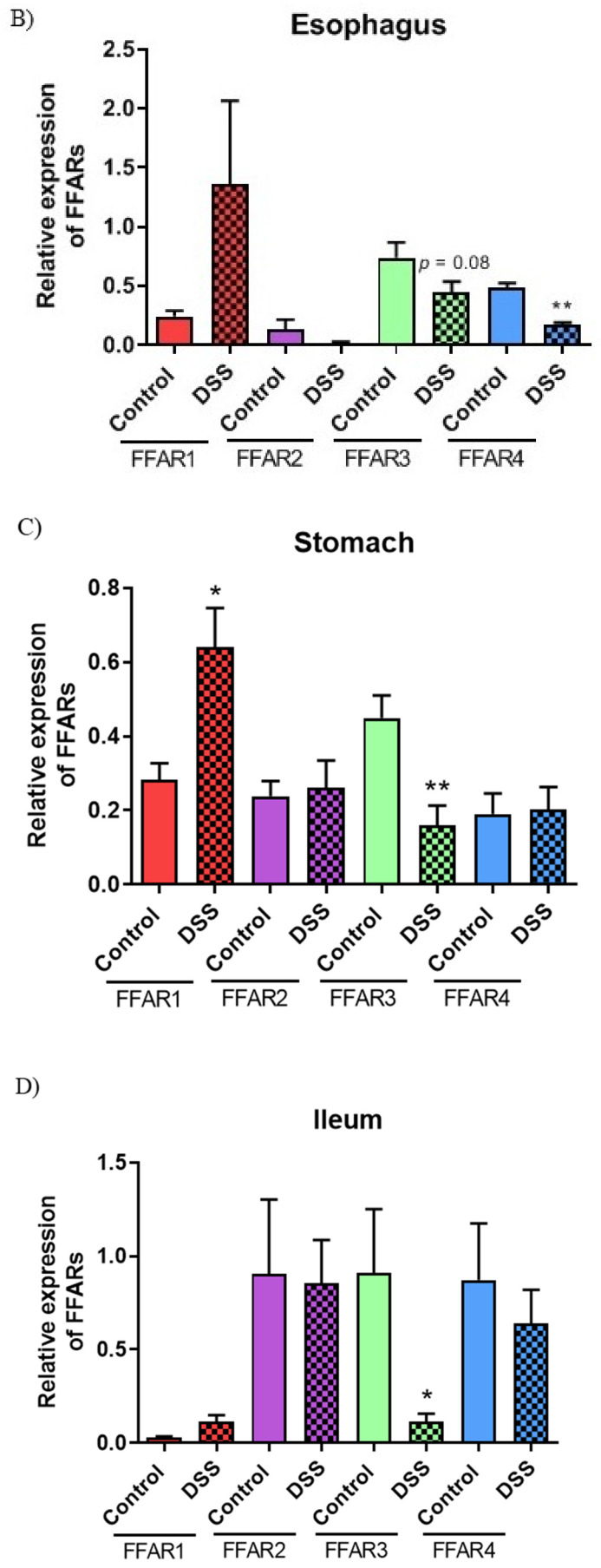

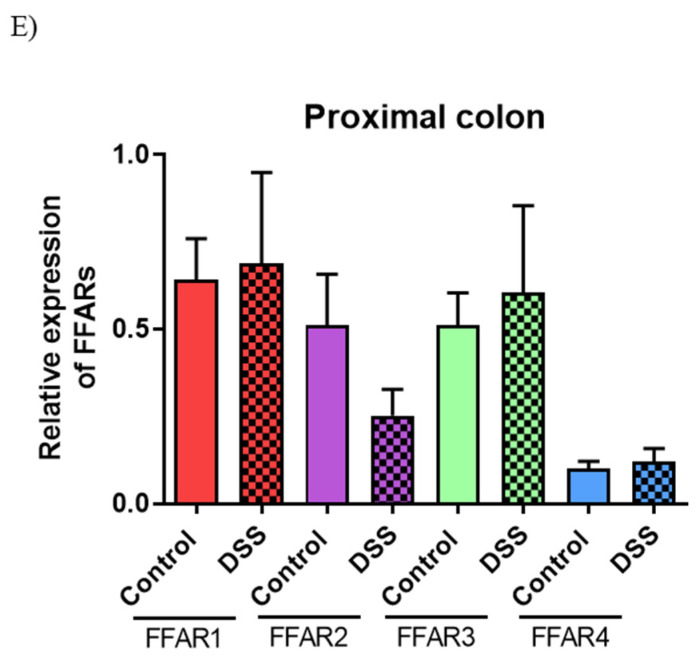
Expression, at the protein level, of FFARs in the various segment of the GI tract. Figure shows data for tongue (**A**), esophagus (**B**), stomach (**C**), ileum (**D**) and proximal colon (**E**). Data represent mean ± SEM of *n* = 5–6 animals per group. Data were analyzed with Student’s *t*-test * *p* < 0.05, ** *p* < 0.01, as compared to control group. DSS: dextran sodium sulfate.

Analysis of FFAR expression in other parts of the GI tract revealed no changes in the tongue (Figure A1A), elevation of FFAR1 and decrease of FFAR3 and 4 in the esophagus (Figure A1B), increase of FFAR1, and decrease of FFAR3 in the stomach (Figure A1C), decrease of FFAR3 in the ileum (Figure A1D), and no alterations in the proximal colon (Figure A1E).

**Figure 5:**
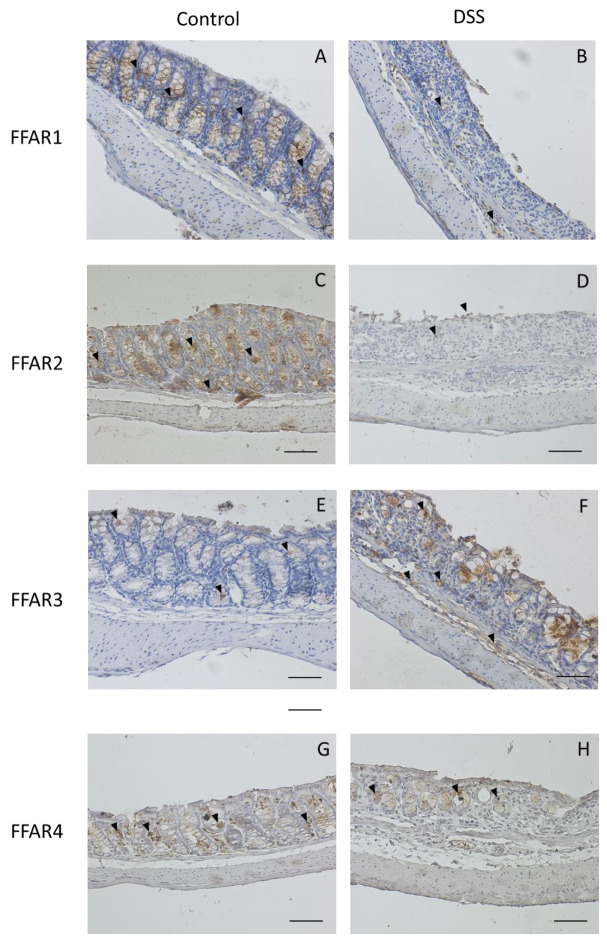

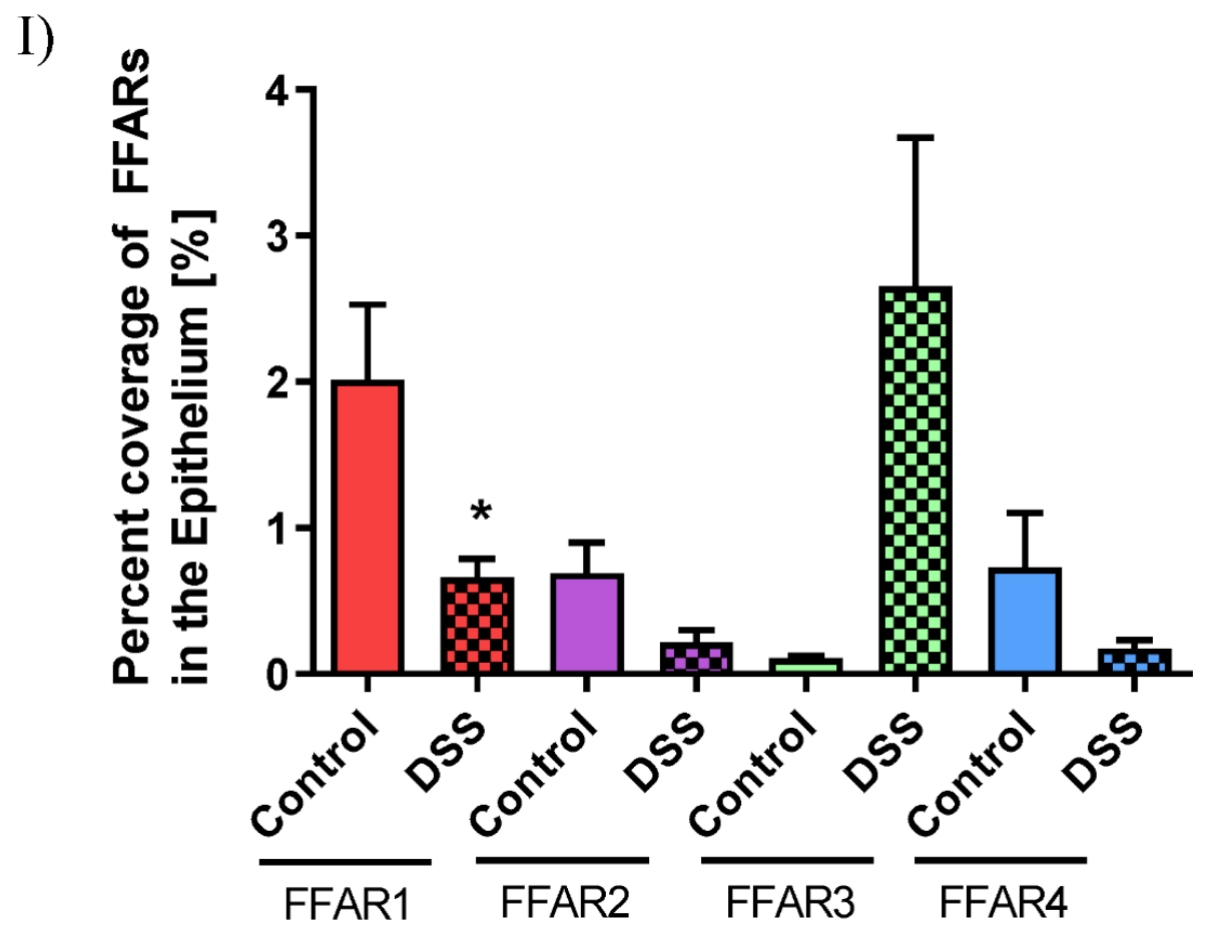
Representative micrographs of immunohistochemical staining of FFARs in formalin-fixed, paraffin-embedded sections of healthy and DSS-treated mouse colon. Figure shows data for FFAR1 (**A**,**B**), FFAR2 (**C**,**D**), FFAR3 (**E**,**F**), and FFAR4 (**G**,**H**). Arrowheads indicate stained cells. Scale bar = 100 µm. The amount of FFARs (**I**) was presented as an epithelium surface area coverage (%). The pictures were taken in bright field setup with standard color charge-coupled device (CCD) camera (Zeiss AxioCam ICc1; Carl Zeiss, Poznan, Poland). Data are presented as means (bars) with SEM. Significance estimated with the use of unpaired *t*-test with Welch’s correction *n* = 2–8; * *p* < 0.05, as compared to control group.

To further characterize the expression of FFAR in the healthy and inflamed colon, we used IHC staining. We observed that in healthy animals, FFAR1 and 2 are predominantly expressed in the colonic crypts, and this expression pattern was no longer visible after treatment with DSS, as the colonic architecture was highly disrupted (Figure 5A–D).

Moreover, in DSS-treated mice, FFAR1-positive cells were visible in both mucosal and submucosal layers of the colonic wall, whereas cells expressing FFAR2 tended to be closer to the luminal site of the colon (Figure 5B,D).

On the other hand, we observed higher number of FFAR3-positive cells in mucosal and submucosal layers of the inflamed colon as compared to the control, where FFAR3 was present in the colonic crypts (Figure 5E,F).

FFAR4 was detected in the colonic crypts of both control and DSS-treated animals, although the damage of mucosal architecture in the latter group caused entrapment of FFAR4-positive cells in the lumen of the disrupted crypts (Figure 5G,H).

Quantification of FFAR expression based on the IHC staining showed significantly decreased presence of FFAR1 and clear, although not significant, increase in the presence of FFAR3 in DSS-treated mice as compared to controls (Figure 5I).

**Figure 6:**
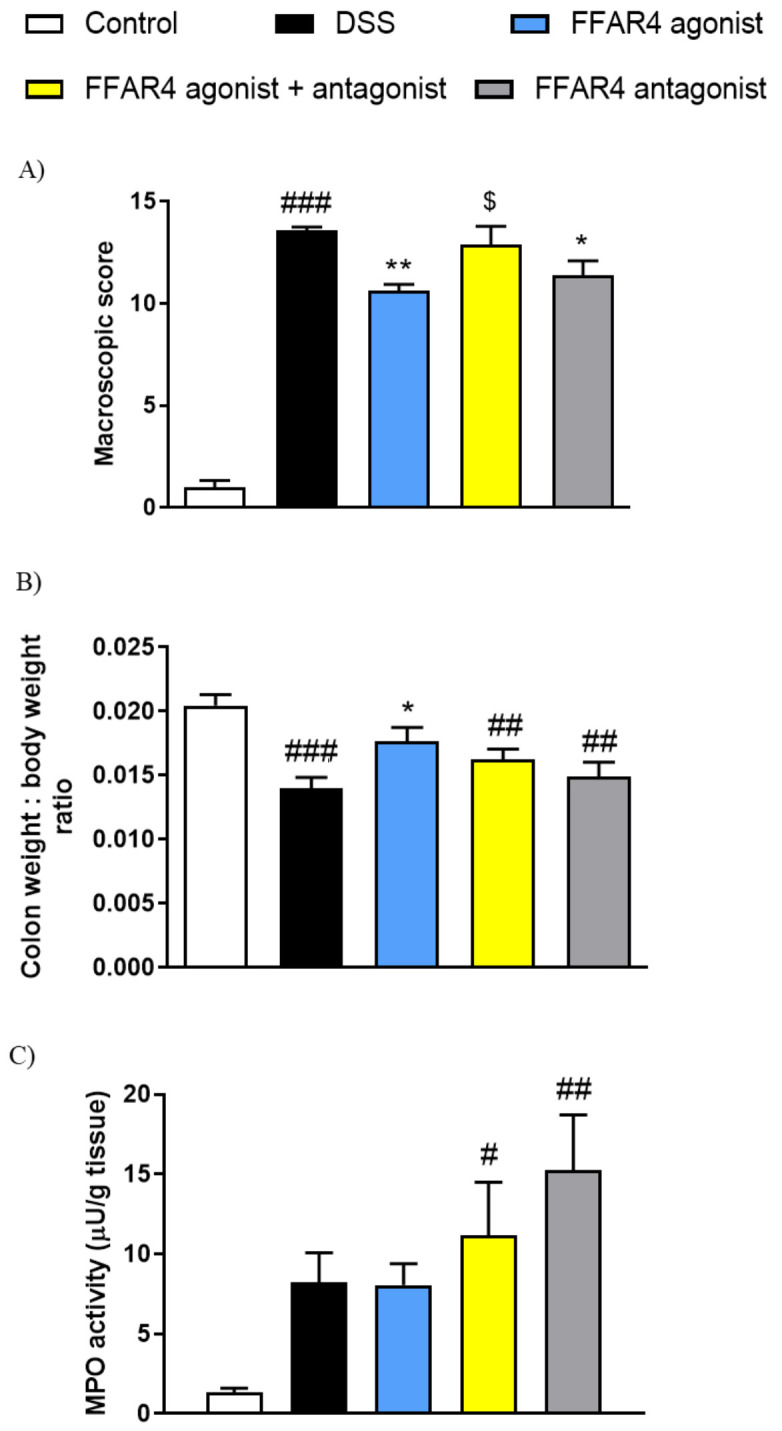
Anti-inflammatory effect of FFAR4 agonist GSK137647 at the dose of 1 mg/kg bw, i.p. twice daily is blocked by pretreatment with FFAR4 antagonist AH7614 (5 mg/kg bw, i.p., twice daily). Figure shows data for macroscopic damage score (**A**), colon weight: body weight ratio (**B**), and MPO activity (**C**). Data represent mean ± SEM of *n* = 8–9 animals per group. Data were analyzed with one-way ANOVA, followed by Newman–Keuls post-hoc test. ^#^ *p* < 0.05, ^##^ *p* < 0.01, ^###^ *p* < 0.001 as compared to control group; * *p* < 0.05, ** *p* < 0.01 as compared to DSS-treated group; ^$^ *p* < 0.05 as compared to FFAR agonist.

To characterize the mechanism of anti-inflammatory action of FFAR4 agonist GSK137647 in the DSS model of colitis, we used a selective FFAR4 antagonist AH7614. As shown in Figure 6A, macroscopically, the action of FFAR4 agonist GSK137647 was reversed by the FFAR4 antagonist AH7614 (5 mg/kg bw, i.p.). Moreover, treatment with the antagonist blocked the beneficial effect of FFAR4 agonist GSK137647 on the relative colon weight (Figure 6B).

Administration of AH7614 caused increase in the MPO activity in the inflamed tissue compared with the control (Figure 6C).

### 3.4. FFAR Agonists Affected the Colonic Epithelial Ion Transport in Healthy and Inflamed Gut

**Figure 7:**
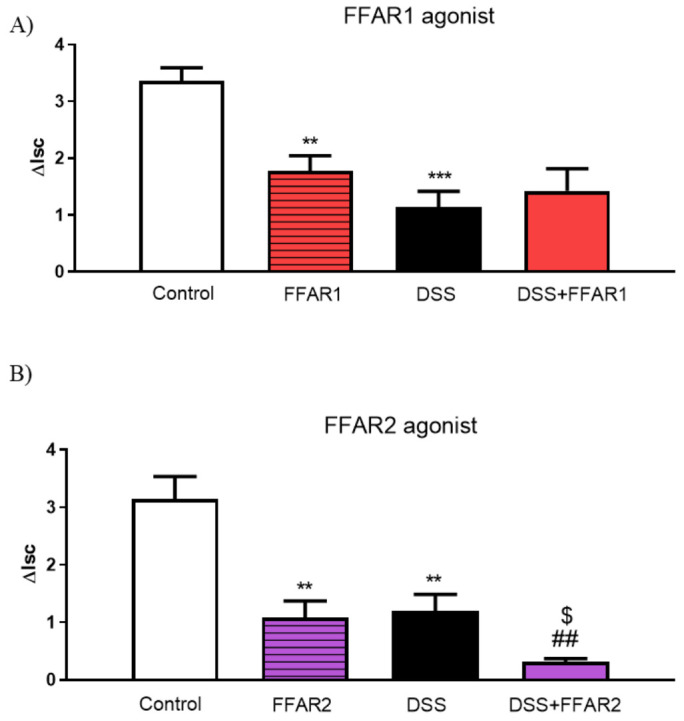

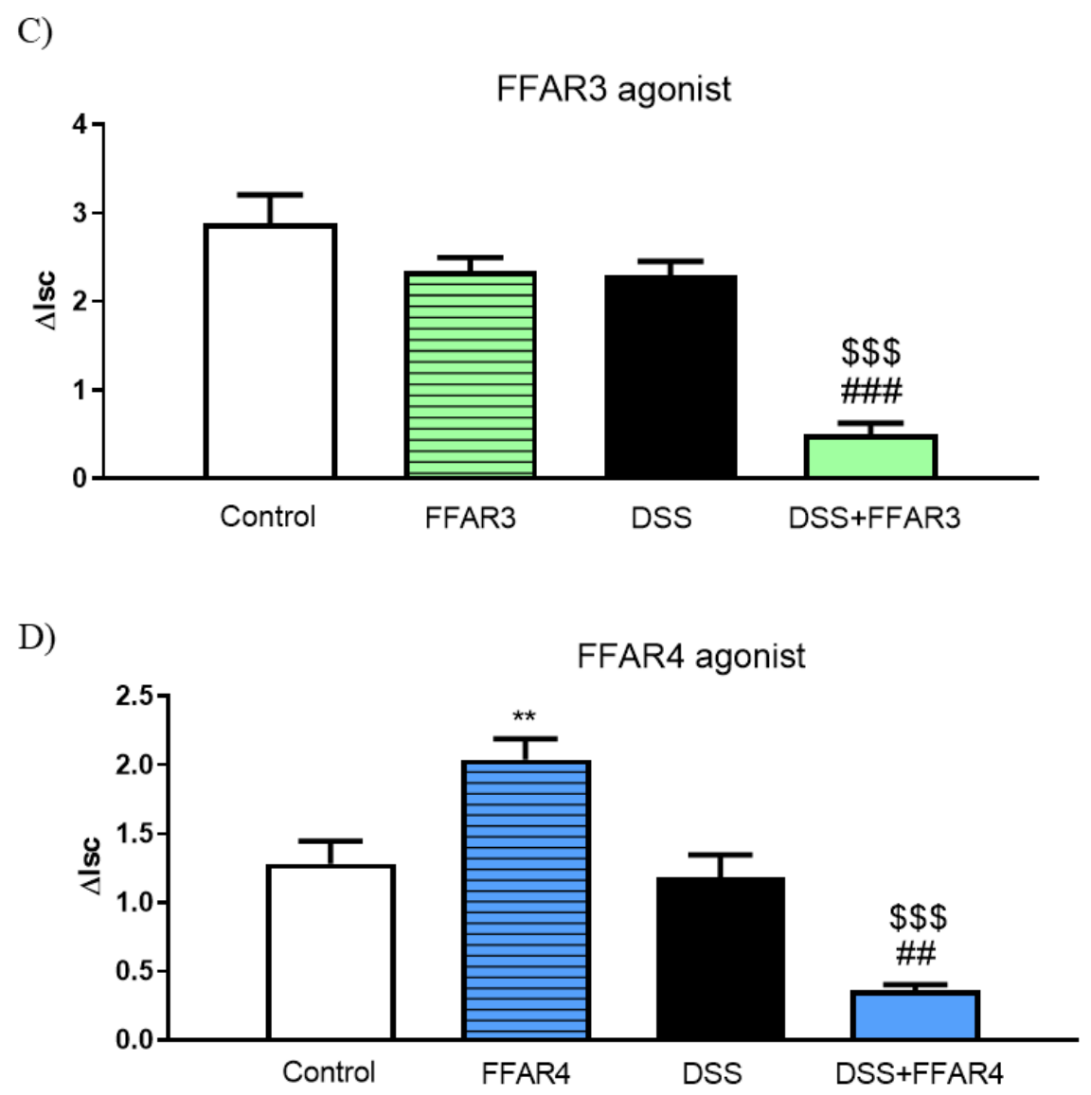
Changes in forskolin-stimulated short-circuit current (ΔIsc) in the mouse colon after basolateral application of (**A**) FFAR1, (**B**) FFAR2, (**C**) FFAR3, and (**D**) FFAR4 agonist (10^−5^ M). Data represent mean ± SEM, *n* = 5. Data were analyzed with one-way ANOVA, followed by Newman–Keuls post-hoc test ** *p* < 0.01, *** *p* < 0.001 as compared to control; ^$^ *p* < 0.05, ^$$$^ *p* < 0.001 as compared to respective FFAR agonist; ^##^ *p* < 0.01, ^###^ *p* < 0.001 as compared to DSS.

First, we investigated the effect of FFAR agonists on cAMP-dependent ion transport in isolated mouse colon mounted in the Ussing chambers. Treatment with DSS caused reduction in the FSK-induced ΔIsc values as compared to controls (Figure 7).

FFAR1 agonist significantly decreased cAMP-dependent transport in control group but did not affect the inflamed mouse colon (Figure 7A).

Incubation with FFAR2 agonist significantly reduced ΔIsc in both healthy and DSS-treated mice (Figure 7B).

Adding FFAR3 agonist to the basolateral side of the colonic tissue only changed the ion flow in the samples from mice treated with DSS (Figure 7C).

FFAR4 agonist did not cause significant differences in either control or DSS groups (Figure 7D).

**Figure 8:**
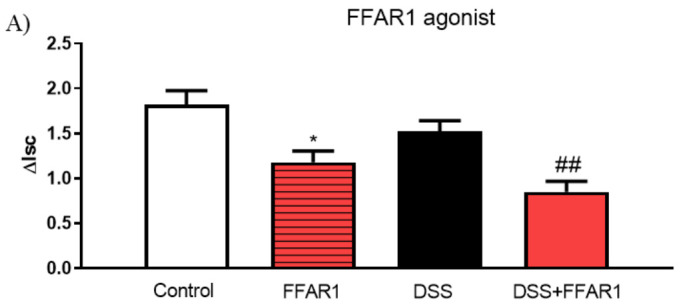

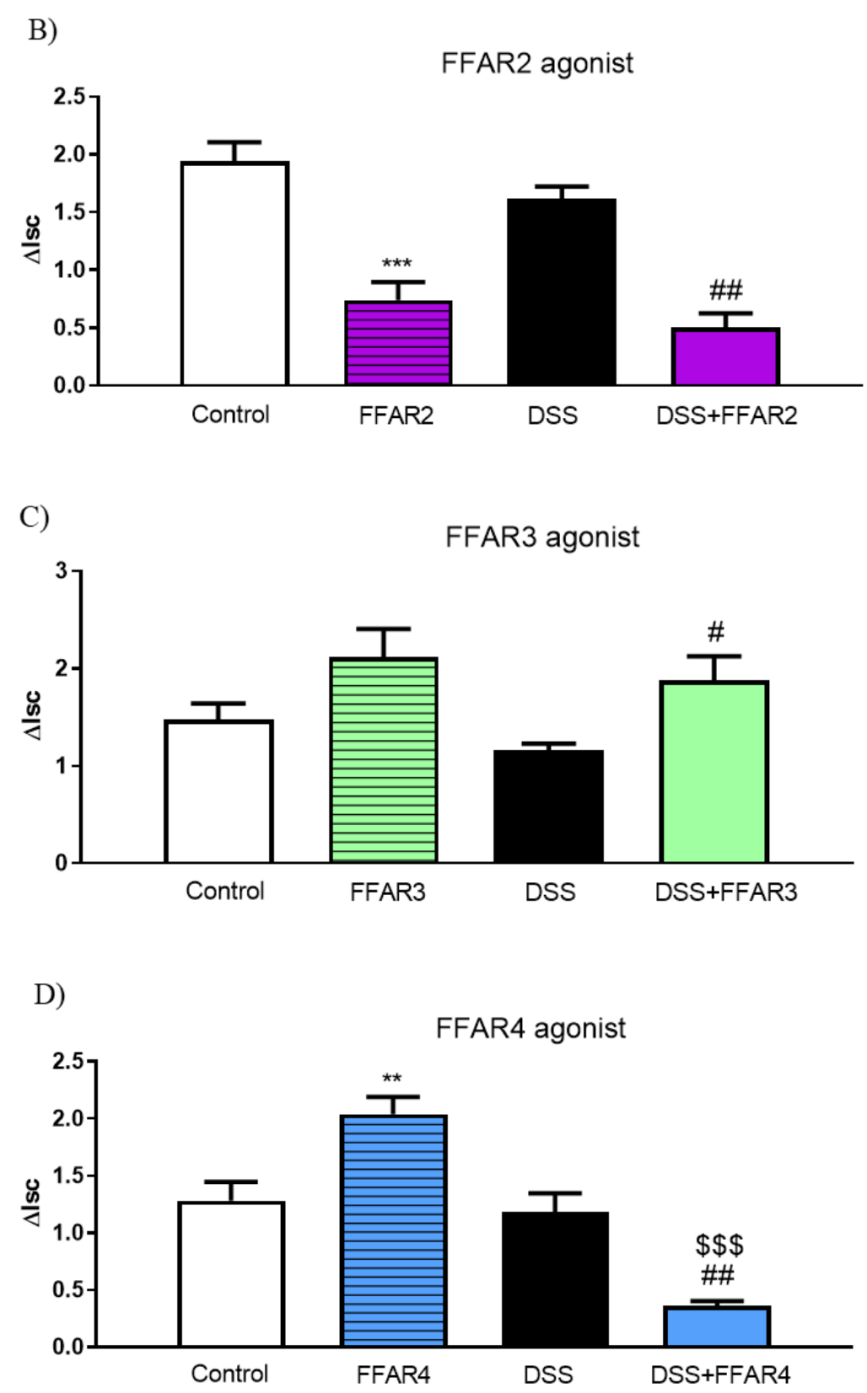
Changes in veratridine-stimulated short-circuit current (ΔIsc) in the mouse colon after basolateral application of (**A**) FFAR1, (**B**) FFAR2, (**C**) FFAR3, and (**D**) FFAR4 agonist (10^−5^ M). Data represent mean ± SEM, *n* = 5. Data were analyzed with one-way ANOVA, followed by Newman–Keuls post-hoc test. * *p* < 0.01, ** *p* < 0.01, *** *p* < 0.001 as compared to control; ^$$$^ *p* < 0.001 as compared to respective FFAR agonist; ^#^ *p* < 0.05, ^##^ *p* < 0.01 as compared to DSS.

To examine ion flow dependence on voltage-dependent Na^+^ channel, we used VER. FFAR1 and FFAR2 agonists significantly lowered, while FFAR3 and FFAR4 ligands increased ion transport in control tissues challenged with VER (Figure 8). Incubation of inflamed tissue with FFAR1, FFAR2, and FFAR4 agonists significantly reduced the ion flow (Figure 8A,B,D), while FFAR3 increased this parameter (Figure 8C).

### 3.5. FFAR4 Agonist Restored Intestinal Permeability Ex Vivo

**Figure 9:**
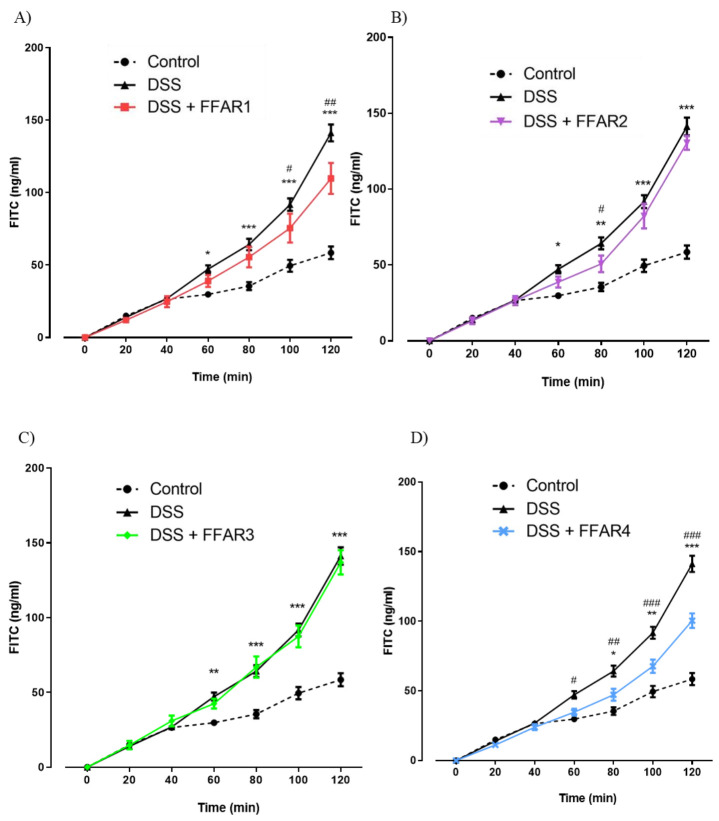
Effect of (**A**) FFAR1, (**B**) FFAR2, (**C**) FFAR3, and (**D**) FFAR4 agonist administration (1 mg/kg bw, twice daily; i.p.) on colon permeability in DSS-treated mice. Data represent mean ± SEM, *n* = 5. Data were analyzed with one-way ANOVA, followed by Newman–Keuls post-hoc test. * *p* < 0.01, ** *p* < 0.01, *** *p* < 0.001 as compared to control; ^#^ *p* < 0.05, ^##^ *p* < 0.01, ^###^ *p* < 0.001 as compared to DSS.

We used fluorescent probe (FITC-labeled dextran) to evaluate the paracellular permeability in mouse colon segments ex vivo. Permeability of colonic tissue to FITC-labeled dextran was increased by DSS at 60–120 min after the addition of the probe to the mucosal chamber. Treatment with FFAR1 agonist significantly weakened the effect of DSS on the permeability of the colonic tissue at 100 and 120 min after the addition of the FITC dextran (Figure 9A).

No differences caused by FFAR2 and FFAR3 agonists were observed (Figure 9B and Figure 2C).

FFAR4 had the strongest effect on the tissue, and the statistical differences with inflamed tissue were observed from 60 min (Figure 9D).

**Figure 10:**
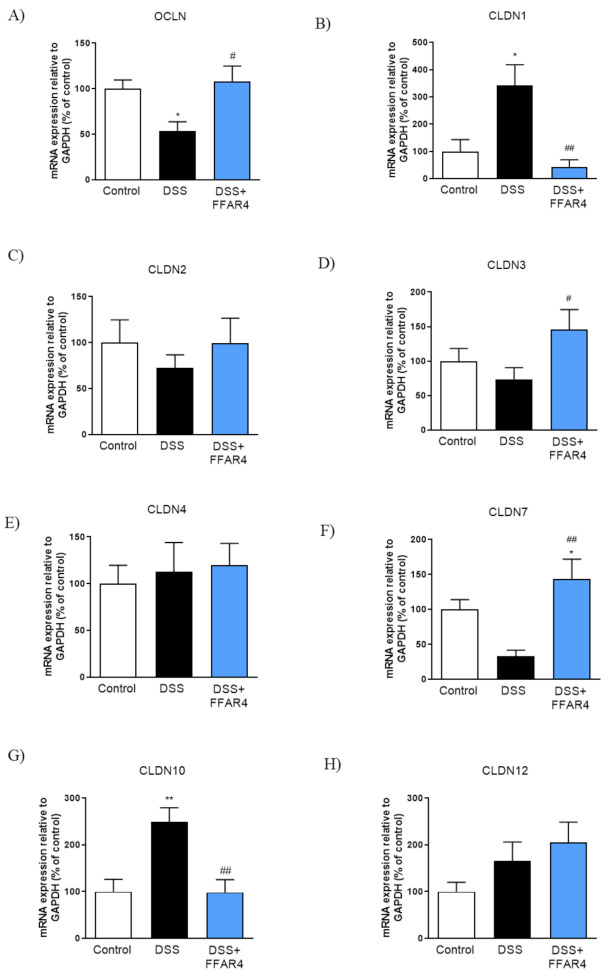
Changes in tight junction-related genes expression in the colon of DSS- and DSS+FFAR4-treated mice. Figure shows data for the expression of occludin (**A**), claudin 1 (**B**), claudin 2 (**C**), claudin 3 (**D**) claudin 4 (**E**), claudin 7 (**F**), claudin 10 (**G**), and claudin 12 (**H**). Values expressed as percent of control group. Data represent mean ± SEM, *n* ≥ 12. Data were analyzed with one-way ANOVA, followed by Newman–Keuls post-hoc test. * *p* < 0.05, ** *p* < 0.01 as compared to control; ^#^ *p* < 0.05, ^##^ *p* < 0.01 as compared to DSS.

Next, we assessed TJs mRNA expression with qPCR using distal colon samples collected from control, DSS-treated, and DDS+FFAR4-treated mice. As shown in Figure 10, the expression of OCLN, CLDN1, CLDN2, CLDN7, and CLDN10 was changed in inflamed tissues in comparison to controls. FFAR4 restored the expression of OCLN, CLDN1, CLDN2, and CLDN10 (Figure 10A–C,G). Expression of CLDN7 was upregulated after treatment with FFAR4 agonist as compared to both control and DSS-treated animals (Figure 10F).

## 4. Discussion

Given the increasing incidence of IBD in both developing and developed regions (322–505 per 100,000 persons in Europe) of the world, with growing prevalence among children, the search for novel treatment strategies is crucial [2]. On the other hand, it is well recognized that a so-called Western diet, rich in fats, contributes to changes in intestinal microbiota and development of IBD [25]. From this perspective, FFARs are attractive drug targets for combating intestinal diseases, as they have protective and anti-inflammatory functions. However, the existing body of literature does not provide clear-cut answer as to how colonic inflammation affects the expression of FFARs, which FFAR agonists exhibit the most potent anti-inflammatory effect, which are able to restore intestinal barrier function, and overall, which member of the FFAR family is the most promising pharmacological target for anti-IBD compounds. Here, we addressed these open questions and revealed that FFAR4 agonist GSK137647 exhibits the most favorable anti-inflammatory properties in vitro and in vivo.

Our in vitro studies showed that agonists of FFAR1, 3, and 4 reduced NO production by immortalized mouse macrophages without affecting their viability. A similar effect was previously shown for n-3 FAs, which exhibited anti-inflammatory effect through FFAR4 and downstream β-arrestin-2 pathway [26]. In addition, we observed a reduction in the expression of SCFA-activated FFAR2 and 3 after treatment with LPS. In the next phase of the in vitro studies, we used cells of intestinal epithelial origin (Caco-2) and mimicked the conditions that occur in the intestinal microenvironment during colitis by addition of TNFα, IL1-β, INFγ, and LPS. Such stimulation induces secretion of IL-6, a pro-inflammatory cytokine commonly observed at high levels in animal models and in IBD patients [27,28]. This experiment revealed that only FFAR4 agonists moderately but significantly downregulated IL-6 production. Moreover, pro-inflammatory cytokines lowered the expression of FFAR1, 2, and 4. This, together with studies on RAW264.7 macrophages, indicates the differential action of FFAR agonists on the immune and epithelial cells during inflammation. FFAR4 agonist exhibited the most favorable anti-inflammatory properties, as it acted on both types of cells.

Here, for the first time, we tested the potential therapeutic effect of the treatment with single FFAR agonists in mouse models of colitis. In the TNBS model, the therapeutic effect of FFAR agonists was not clear due to the high variability of the results, which may be caused by the lot of TNBS and strain of animals used in the study, which is often observed in this model, hence the lack of statistical significance. Nevertheless, FFAR4 exhibited the most favorable effect on the macroscopic and biochemical parameters of inflammation. Furthermore, we observed a robust increase in FFAR1 expression, with no apparent change in FFAR2–4 during TNBS-induced colitis. Our findings obtained in the DSS model of colitis confirmed that stimulation of FFAR4 significantly alleviated macroscopic parameters of colitis, with no apparent effect on the MPO activity, which suggests weak or perhaps lack of the effect on neutrophil infiltration. Interestingly, the expression of FFAR1 increased during DSS-induced inflammation not only in the distal colon but also in the esophagus and stomach, suggesting increased response to LCFAs in these segments of the GI tract. However, the role of FFAR1 signaling in colitis remains unclear given the lack of effect elicited by the agonist of this receptor.

One of the issues associated with IBD that may be related to the fact that the modern diet leads to impaired permeability of the gut-blood-barrier, which leads to a variety of symptoms, including diarrhea, abnormal motility, and increased immune response to antigens penetrating the epithelium. Here, we addressed the role of FFARs in this phenomenon. First, we examined the effect of FFAR agonist on FSK-induced epithelial ion transport. FSK is a diterpene useful in studying the mechanism underlying secretory diarrhea. It activates the catalytic subunit ofadenylate cyclase and strengthens cAMP-dependent signaling [29]. cAMP, which acts as a secondary messenger, activates protein kinase A (PKA), leading to increase in Cl^−^ and water secretion into the gut lumen through cystic fibrosis transmembrane conductance regulator (CFTR) channels [30]. To the best of our knowledge, this is the first study in this field. It was previously reported that in DSS-induced colitis, the ion transport is suppressed [31]. Similarly, in our study, tissues collected from mice treated with DSS had reduced ion transport compared to the control group. In our study, pre-incubation of control tissue with FFAR1 and 2 agonists induced DSS-like effect (reduction of ∆Isc). In inflamed tissue, activating all FFARs except for FFAR1 strongly reduced ∆Isc. Following the results, we could assume that not the activation but rather blocking of these receptors might be a good direction in the design of new anti-diarrheal therapeutic strategies. Activation of the FFAR1 did not significantly influence the ion transport in the DSS group, which may be caused by too low concentration of the agonist used in this study. Hence, this receptor should also be considered in the future, as other concentrations or different agonists could increase its impact.

In addition, we tested the effect of FFAR agonists on VER-induced ion flow. VER leads to depolarization of enteric neurons through elevated voltage-sensitive Na^+^ permeability and subsequently causes epithelial Cl^−^ secretion across the colonic mucosa [32]. Thus, this compound is used to investigate the effect of neural stimulation on the intestinal barrier. Here, DSS suppressed the VER-induced ∆Isc values to the lower extent than in tissues stimulated with FSK. Both FFAR1 and FFAR2 agonists significantly reduced the ion transport in control and inflamed colons. FFAR4 agonist acted differently in the controls, where it elevated the current, and the DSS-treated group, where it strongly inhibited the ∆Isc. Interestingly, stimulation of FFAR3 increased ∆Isc in both groups, becoming the potential strategy for the treatment of diarrhea symptoms among UC patients. As this receptor is activated by SCFAs derived from the fermentation of dietary fibers by intestinal bacteria, the diet enriched by those FAs should be taken under consideration regarding UC patients. Additionally including products containing LCFAs, natural ligands for FFAR4 may have anti-secretory properties but only in the healthy group. Consequently, both diets are an interesting direction in preventing the disease symptoms among people in the risk group. Moreover, it was previously shown that a diet enriched with oleic acid (LCFA) in DSS model of colitis delayed diarrhea and rectal bleeding but did not improve colonic histopathology compared to controls [33]. It was also shown that docosahexaenoic acid (LCFA) reduced infiltration of inflammatory cells, lowered inflammation scores, decreased pro-inflammatory cytokines, and improved body weight in the model of colitis induced by IL-10 deficiency [34]. On the other hand, many studies present exacerbation of the disease activity in rodents and humans with chronic docosahexaenoic acid treatment [35,36,37]. It is somehow consistent with our study that stimulation of the FFAR1 and FFAR4 seemed to worsen the epithelial ion transport in the DSS group, which may lead to increase in diarrhea.

Then, we investigated the effect of FFAR agonists on changes in the colon permeability caused by colonic inflammation. We observed a significant increase in the amount of FITC that passed through the inflamed colon as compared to the control. FFAR4 agonist reversed DSS action to the greatest extent, suggesting its potential in alleviation of UC symptoms caused by increase in intestinal epithelial barrier permeability.

The supposed contradiction in the effect of FFAR4 agonist on epithelial ion transport and gut permeability may be explained by different experimental setups both in terms of molecular mechanisms and method of drug delivery. Measurement of ΔIsc included the influence of additional factors, namely FSK and VER, that are not present in the FITC experiment. Moreover, the ion transport was assessed after pre-incubation of the tissue with FFAR agonist that lasted 15 min, while in the permeability experiments, the FFAR4 agonist was administered i.p. for 4 days in a row before the measurement. Short time of the tissue being exposed to an agonist (in the ΔIsc assessment) and treating mice with FFAR4 agonist simultaneously with DSS administration (in the permeability study) may be crucial. Interestingly, both effects are significant, which creates potential for further investigations.

The GI epithelial barrier plays an important role in separation of the inside of the body from the outside environment. TJs not only maintain cell polarity but are also associated with regulation of differentiation of the intestinal epithelium. Both loss and gain-of-function studies in mice have revealed specific roles of the tight junctions (TJ) in barrier function or selective ion permeability [38]. Mice with overexpression of CLDN1 are more prone to colitis and demonstrate slower recovery following the DSS treatment [39]. Up-regulation of CLDN2 in the mouse GI tract showed its importance in regulation of epithelial permeability, inflammation, and proliferation [40]. CLDN7 is essential for proper functioning of the gut since knockout of this protein caused serious intestinal defects, including mucosal ulcerations and inflammation, which led to death [41]. Jin et al. did not find differences in the expression of CLDN1 and CLDN2 in the distal colon of inflamed mice as compared with control group. On the other hand, OCLN expression was reduced in mice treated with DSS [42]. It was also reported that that expression of OCLN and CLDN3 is reduced in DSS-induced colitis compared to the control group [43]. Based on our results, we also decided to investigate the effect of FFAR4 agonist on TJs mRNA expression in the setting of colonic inflammation. FFAR4 restored the expression of OCLN, CLDN1, CLDN2, and CLDN10. Similar results were also obtained with the administration of walnut oil, which is believed to activate FFAR4 [22].

In view of the above, we propose a new approach in IBD treatment based on the use of FFAR agonists. The novelty of our study has several aspects. The potential anti-inflammatory effect caused by activation of individual FFARs has not been assessed before. Here, we demonstrated that FFAR4 (natural sensor of LCFAs) exhibits the strongest anti-inflammatory activity both in vitro and in vivo. Moreover, this is the first study showing the beneficial effect of FFAR4 activation on intestinal barrier function and expression of TJ proteins. In this study, we used synthetic FFAR agonists as pharmacological tools to trigger FFAR-dependent responses. This choice was dictated by well-characterized, pharmacological properties of these compounds and ease of their administration. However, positive validation of the idea of employing FFAR agonists to combat intestinal inflammation can, perhaps, be extrapolated to FFAR activators of natural origin, namely FFAs. Consequently, we envisage anti-IBD interventions based on nutrients especially for those who prefer to refrain from synthetic drugs yet choose the treatment that provides similar efficacy. In line, we previously showed that treatment with a diet enriched with walnut oil, rich in FFAR4 agonists, such as α-linolenic acid and high n-3/n-6 omega FFAs ratio, alleviates intestinal inflammation in mice [22]. Colitis was also improved by fish oil, rich in n-3 LCFA, in two other studies [44,45]. Moreover, it was shown that eicosapentaenoic acid administered to UC patients in remission (partial Mayo score < 2, fecal calprotectin ≥ 150 µg/g) yielded higher rates of 100-point reduction in calprotectin levels and maintenance of clinical remission [46]. On the other hand, in CD, such effect has not been observed. Patients with quiescent CD (CDAI < 150) supplemented with 4 g of n-3 FFAs daily experienced relapse of the disease at the same rate as those given placebo, which indicates the differential influence of nutrients acting as FFAR4 agonists in various types of gut inflammation [47].

## 5. Conclusions

Notably, one of the suggested forms of taking natural FFAR ligands is via a diet. The advantage of that form arises from much easier intake, therefore perhaps increasing patients’ compliance as well as lessening production costs. The outcomes of the first two phases of clinical trials assessing the effects of FFAR2 antagonist showed that this molecule does not cause side effects, and its administration is well tolerated. However, phase III study showed the lack of efficiency (ClinicalTrials.gov Identifier: NCT00311610) [48]. Finally, IBD as chronic, relapsing inflammatory entities elevate the risk of large bowel cancer development. Thus, by treating the disease with FFAR ligands, we could lower this risk [49,50,51].

The fact that the data obtained for stimulation/blockade of FFAR may be slightly inconsistent derives from the pleiotropic effects of FFAR ligands connected with receptor distribution and miscellaneous signaling pathways. Perhaps an orchestrated stimulation of two or three types of FFARs could be the most effective way to alleviate inflammation in vivo. Such hypothesis could be verified in future studies aimed at exploring the potential of FFARs in IBD.

## 6. Study Limitations

Although TNBS- and DSS-induced colitis in mice are commonly used preclinical models of colitis, there are some limitations due to the fact that chemical injury does not fully simulate the chronically inflamed environment in human colon in IBD. Therefore, considering the results derived from this research, further investigation is still needed to explore the effect on both activation and blocking the FFAR receptors and to fully evaluate the therapeutic value of these receptors as targets in colitis.

## Data Availability

Data available, upon reasonable request, from the corresponding author.
